# Down regulation of RNA binding motif, single-stranded interacting protein 3, along with up regulation of nuclear HIF1A correlates with poor prognosis in patients with gastric cancer

**DOI:** 10.18632/oncotarget.13605

**Published:** 2016-11-25

**Authors:** Youliang Wu, Dapeng Yun, Yingjie Zhao, Yuqi Wang, Ruochuan Sun, Qiang Yan, Shangxin Zhang, Mingdian Lu, Zhen Zhang, Daru Lu, Yongxiang Li

**Affiliations:** ^1^ Department of General Surgery, The First Affiliated Hospital of Anhui Medical University, Hefei, People's Republic of China; ^2^ State Key Laboratory of Genetic Engineering and MOE Key Laboratory of Contemporary Anthropology, School of Life Sciences, Fudan University, Shanghai, People's Republic of China

**Keywords:** gastric cancer, RBMS3, HIF1A, MVD, prognostic marker

## Abstract

Frequent loss of multiple regions in short arm of chromosome 3 is found in various tumors including gastric cancer (GC). RNA binding motif, single-stranded interacting protein 3 (RBMS3) is a tumor suppressor gene located in this region and mediates cancer angiogenesis. However, the role of RBMS3 in GC remains unclear.

To evaluate whether RBMS3, together with HIF1A, another key regulator of angiogenesis, predicts GC prognosis, the levels of RBMS3 and HIF1A were first examined by quantitative PCR (qPCR) and western blot from 27 fresh frozen GC and paired normal gastric tissues and then tested by immunohistochemistry (IHC) from 191 GC and 46 normal controls. Moreover, uni- and multivariate analysis were employed to assess the correlations between their levels and microvessel density (MVD) and clinical prognosis. To further identify RBMS3 function *in vitro*, cell proliferation assay, clonogenic assay, flow cytometry analysis and endothelial cell tube formation assay were employed.

We found that RBMS3 level was decreased, whereas HIF1A was elevated in GC. Furthermore, we demonstrated that RBMS3 was an independent prognostic factor and the levels of RBMS3 and HIF1A were associated with GC angiogenesis and histopathological differentiation: patients with lower RBMS3 level and higher nuclear HIF1A expression had poorer prognosis. Besides, gain- and loss-of-function study revealed RBMS3 regulation on G1/S progression, cell proliferation and the tube formation of human umbilical vein endothelial cells (HUVECs) *in vitro.* These findings implicated that RBMS3 and nuclear HIF1A could act as prognostic biomarkers and therapeutic targets for GC.

## INTRODUCTION

Gastric cancer (GC) is one of the most lethal malignancies around the world, resulting in 951,600 new stomach cancer cases, and 723,100 deaths in 2012 worldwide, specifically in eastern Asian countries [1]. Moreover, most of the GC patients have already been in advanced stages with poor prognosis when diagnosed due to insufficient early specific symptoms. Thus, finding out more valuable molecular prognostic biomarkers for GC is imperative.

Deletion of multiple regions on the short arm of chromosome 3 (3p) is one of the most frequent genetic alterations in many human solid tumors including GC [2–5], which suggests the importance of the tumor suppressor genes (TSG) inside the region. RNA binding motif, single-stranded interacting protein 3 (RBMS3), located at 3p24-p23, belongs to the c-Myc gene single-strand binding protein (MSSP) family, which regulates DNA replication, gene transcription, cell cycle progression, and apoptosis by interacting with the c-Myc protein [6–8]. It inhibits microvessel formation by down-regulation of MMP2, MMP-9, VEGF and β-catenin [9]. Besides, RBMS3 directly binds to the promoter region of c-Myc in esophageal squamous cell carcinoma (ESCC) [10], and arrests cell cycle at the G1/S checkpoint. Moreover, RBMS3 is reported to reside in the cytoplasm, implying its possible cytoplasmic functions like RNA metabolism control [11]. From the clinical data, despite that down-regulated RBMS3 in lung squamous cell carcinoma (LSCC), nasopharyngeal carcinoma (NPC) and ESCC are found to strongly associate with unfavorable outcome [9, 10, 12], the expression and impacts of RBMS3 on GC progression is still unknown.

HIF-1 (a basic-helix-loophelix-PAS transcription factor) is a heterodimer consisting of HIF-1α (HIF1A) and HIF-1β subunits [13]. It dimerizes with HIF1β and translocates from the cytoplasm to the nucleus, which induces target genes like vascular endothelial growth factor (*VEGF*) [14], a key factor in tumor angiogenesis. The correlation between MVD and HIF1A expression has been found in many cancers [15–17], including GC [18]. Besides, HIF1A is overexpressed in various tumors and its expression is often positively associated with poor prognosis [19]. Furthermore, it is clear that HIF1A expresses in both cytoplasm and nuclei of a cell and exerts different functions in cancer progression [20]. However, whether the difference in subcellular localization of HIF1A correlates with clinical prognosis still remains obscure. We infer that different localization of HIF1A works differently in predicting clinical prognosis, which needs further dissection. In this study, we potentiated the clinical relevance of RBMS3 and different subcellular location of HIF1A by their expression and correlation to GC prognosis. Moreover, we identified the role of RBMS3 in GC proliferation and angiogenesis *in vitro*.

## RESULTS

### Expression of RBMS3 and HIF1A mRNA and protein in fresh tissues

**Figure 1:**
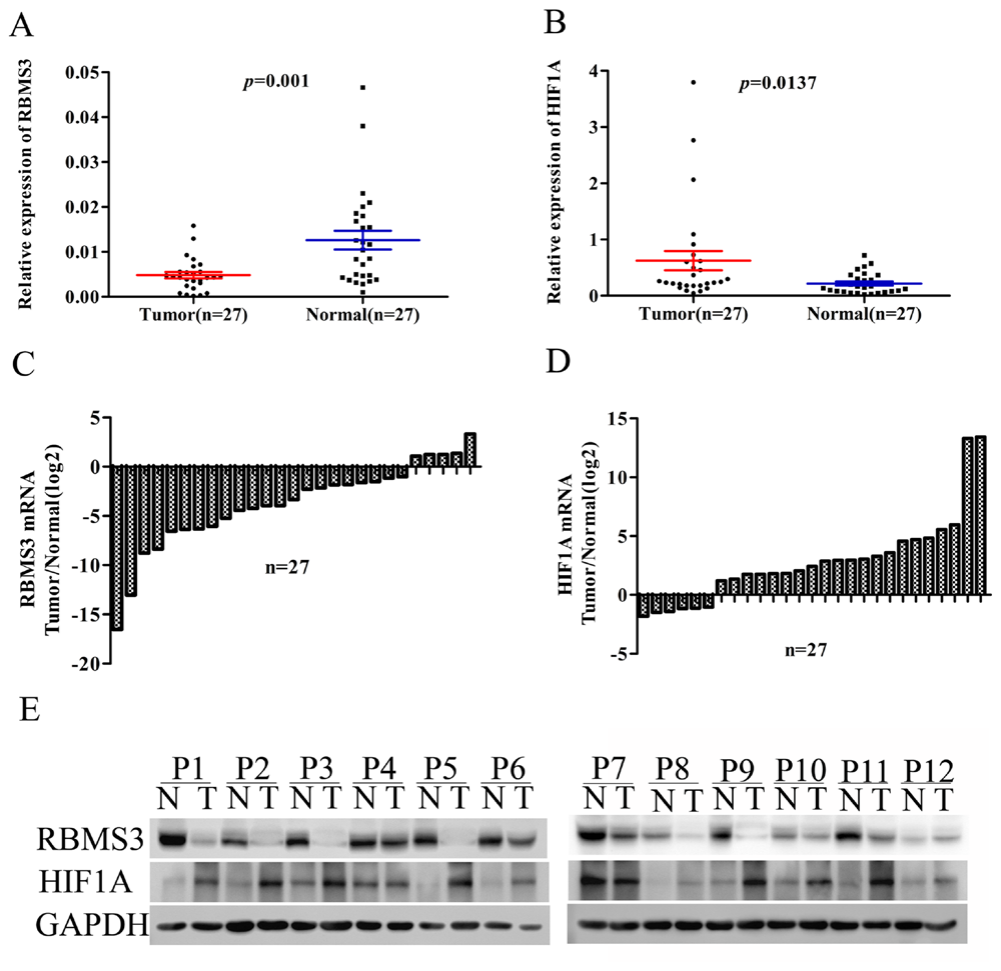
The mRNA and protein level of RBMS3 and HIF1A in clinical samples **A, B.** Scatter plots of the relative expression of RBMS3 (A) and HIF1A (B) mRNA in tumor and normal tissues. **C, D.** Bar plots of RBMS3 (C) and HIF1A (D) expression in GC tissues compared with matched normal tissues. Each patient was presented as the log2 ratio of tumor tissue/normal tissue. **E.** The protein expression levels of RBMS3 and HIF1A were analysed by western blot assay. Representative protein expression level of RBMS3 and HIF1A in 12 pairs of tumor (T) and corresponding normal tissues (N). GAPDH were used as an endogenous control.

Down-regulation of RBMS3 and up-regulation of HIF1A have been reported in some human cancers [10, 19]. However, the correlation between RBMS3 and HIF1A in GC is still unknown. To investigate the relationship between RBMS3 and HIF1A in GC tissues, we performed quantitative PCR (qPCR) and western blot in a cohort of 27 fresh frozen GC and matched normal tissues. We found the mRNA levels of *RBMS3* were markedly lower than matched controls, while *HIF1A* level were significantly elevated (Figure 1A, 1B; *p*=0.001, *p*=0.0137, respectively). When we defined <1-fold change as down-regulation and >1-fold change as up-regulation, it showed that 81.5% (22/27) of GC tissues displayed down-regulated *RBMS3*, and 77.8% (21/27) had up-regulated *HIF1A* (Figure 1C, 1D). To further verify their protein expression, we then performed western blot and as shown in Figure 1E, when compared to matched controls, RBMS3 was decreased while HIF1A was increased in the same cohort. Taken together, these findings confirm that in human GC, RBMS3 is down-regulated, while HIF1A is up-regulated in both mRNA and protein levels.

### Immunostaining for RBMS3 and HIF1A

**Figure 2:**
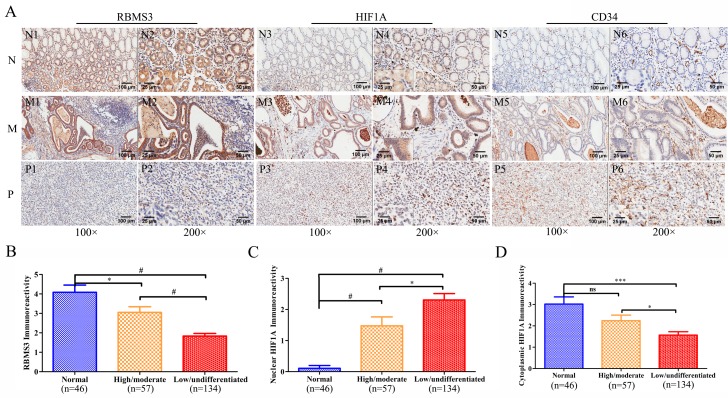
Immunohistochemical staining of RBMS3, HIF1A and CD34 protein in GC and randomly normal gastric tissues **A.** Representative images of RBMS3, HIF1A and CD34 as followings: normal gastric tissues (N) with RBMS3^+^ (N1, N2), cytoplasmic HIF1A^+^ (N3, N4) and low MVD (N5, N6) expression; middle differentiated (M) GC with RBMS3^+^ (M1, M2), cytoplasmic HIF1A^+^ (M3, M4) and low MVD (M5, M6) expression; poor differentiated (P) GC with RBMS3^−^ (P1, P2), nuclear HIF1A^+^ (M3, M4) and high MVD (M5, M6) expression. Magnification: 100× (N1, N3, N5, M1, M3, M5, P1, P3 and P5, bar = 100 μm) and 200× (N2, N4, N6, M2, M4, M6, P2, P4 and P6, bar = 50 μm; insert, 400×, bar = 25 μm). Immunoreactivity scores of RBMS3 **B.**, nuclear HIF1A **C.** and cytoplasmic HIF1A **D.** staining in normal gastric tissues and high/moderate to low/undifferentiated GC histological grade are represented as mean ± SEM. *, *p*<0.05; **, *p*<0.01; ***, *p*<0.001; #, *p*<0.0001.

To further confirm the expression of RBMS3 and HIF1A, we examined their levels by immunohistochemical staining in a validation cohort consisting of 191 patients (Figure 2A). The characteristics of the cohort were summarized in Table 1. For RBMS3, the positive staining was mainly localized in the cytoplasm and exhibited a significant difference: 39.27% (75/191) of the GC samples were positive while 67.39% (31/46) of the normal controls were positive (*p*=0.001). For HIF1A, the percentage of positivity between GC and normal control samples was 67.54% (129/191) versus 45.65% (21/46, *p*=0.006).

**Table 1:** Relationship between RBMS3 and nuclear HIF1A expression and clinicopathological variables (n=191)

Clinicopathological variables	Total	RBMS3 expression		*p* value	nuclear HIF1A expression		*p* value
		positive	negative		positive	negative	
Gender				0.851			0.595
Male	144	56	88		52	92	
Female	47	19	28		19	28	
Age (y)				0.462			0.669
<61	93	39	54		36	57	
≥61	98	36	62		35	63	
Tumor size (cm)				0.926			0.464
<6	123	48	75		43	80	
≥6	68	27	41		28	40	
Differentiation				**0.005**			**0.043**
High/moderate	57	31	26		15	42	
Low/undifferentiated	134	44	90		56	78	
Location				0.417			0.982
Upper	90	31	59		34	56	
Middle	43	18	25		16	27	
Lower	58	26	32		21	37	
Depth of invasion				0.173			0.246
T1/T2	32	16	16		9	23	
T3/T4	159	59	100		62	97	
Lymph node metastasis				0.395			0.883
Yes	136	56	80		51	85	
No	55	19	36		20	35	
TNM				0.28			0.751
I/II	70	31	39		25	45	
III/IV	121	44	77		46	75	

Note: TNM, tumor-node-metastasis; *p* values were detected by the Pearson Chi-square test; *p*<0.05 was defined statistically significant and was given in bold.

**Figure 3:**
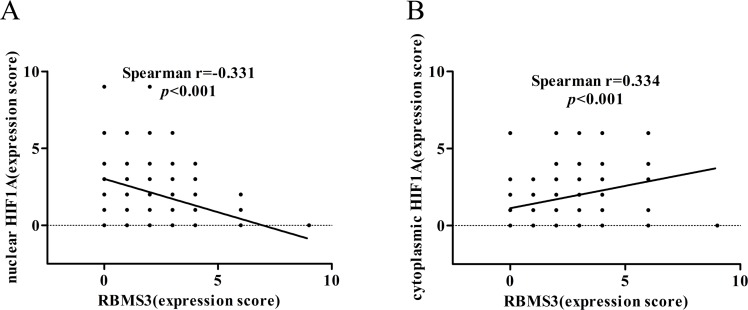
Correlation between RBMS3 and HIF1A levels in GC **A.** The correlation between RBMS3 and nuclear HIF1A levels. **B.** The correlation between RBMS3 and cytoplasmic HIF1A levels.

As HIF1A was reported to express in both cytoplasms and nucleus [21], to evaluate whether its subcellular localization could contribute to the difference in positivity between the two groups, we compared the stainings in nucleus and cytoplasms respectively. Interestingly, we found 37.17% (71/191) of the HIF1A nuclei staining in GC samples were positive, while only 2.2% (1/46) in the normal controls (*p*<0.001). However, we didn't observe any differences in cytoplasmic staining: 35.08% (67/191) in GC samples versus 45.65% (21/46) in normal controls (*p*=0.183). Furthermore, we discovered that RBMS3 levels were negatively correlated with nuclear HIF1A (r=−0.331, *p*<0.001, Figure 3A), but positively correlated with cytoplasmic HIF1A (r=0.334, *p*<0.001, Figure 3B) in the same cohort. In sum, our data show that RBMS3 is inhibited whereas nuclear HIF1A is elevated in GC and that RBMS3 levels are correlated with both cytoplasmic and nuclear HIF1A.

### The relationship between the expression of RBMS3, HIF1A and clinicopathological parameters of GC

In order to illustrate the clinical importance of RBMS3 and HIF1A, we examined the relationship between RBMS3 and HIF1A and clinicopathological parameters of GC patients. TNM tumor stage was grouped into two categories: “early” (I/II) and “advanced” (III/IV). The lesional lymph node stages were classified as lymph node-negative (No) and lymph node-positive (Yes). As shown in Table 1, RBMS3^−^ was also significantly related to poor histopathological differentiation (high/moderate vs low/undifferentiated, *p*=0.005). Compared to normal and high/moderate, the immunoreactivity of RBMS3 was dramatically decreased in low/undifferentiated (Figure 2B). Similarly, nuclear HIF1A^+^ was significantly correlated with poor histopathological differentiation (high/moderate vs low/undifferentiated, *p*=0.043). The immunoreactivity of HIF1A in normal, high/moderate and low/undifferentiated was presented in Figure 2C and 2D. Nuclear HIF1A immunoreactivity in low/undifferentiated was significantly higher than that in normal and high/moderate; cytoplasmic HIF1A immunoreactivity in low/undifferentiated was significantly lower than that in normal and high/moderate. However, the expression of either RBMS3 or nuclear HIF1A had no significant relationship with the other clinicopathological parameters.

Besides, Table 2 showed the clinicopathologic features and RBMS3/nuclear HIF1A expression in patients with GC. We observed that RBMS3^−^/nuclear HIF1A^+^ group showed poorer differentiation than RBMS3^+^/nuclear HIF1A^−^ group in GC samples, which further supported that RBMS3^−^/nuclear HIF1A^+^ GC tumors were more malignant than RBMS3^+^/nuclear HIF1A^−^ tumors. Collectively, our findings indicate that RBMS3^−^ and nuclear HIF1A^+^ expression are correlated with histopathological differentiation.

**Table 2:** RBMS3/nuclear HIF1A expression and clinicopathological variables in GC patients (n=191)

Clinicopathological variables	Total	RBMS3^−^/Nuclear HIF1A^−^	RBMS3^+^/Nuclear HIF1A^−^	RBMS3^−^/Nuclear HIF1A^+^	RBMS3^+^/Nuclear HIF1A^+^	*p* value
		n(63)	n(57)	n(53)	n(18)	
Gender						0.472
Male	144	45	47	38	14	
Female	47	18	10	15	4	
Age (y)						0.649
<61	93	29	28	29	7	
≥61	98	34	29	24	11	
Tumor diameter (cm)						0.337
<6	123	38	42	31	12	
≥6	68	25	15	22	6	
Differentiation						**7.93E-06**
High/moderate	57	11	31	14	1	
Low/undifferentiated	134	52	26	39	17	
Location						0.915
Upper	90	29	27	26	8	
Middle	43	17	10	12	4	
Lower	58	17	20	15	6	
Depth of invasion						0.291
T1/T2	32	9	14	6	3	
T3/T4	159	54	43	47	15	
Lymph node metastasis						0.716
Yes	136	47	38	37	14	
No	55	16	19	16	4	
TNM						0.091
I/II	70	17	28	19	6	
III/IV	121	46	29	34	12	

Note: *p*<0.05 was defined statistically significant and was given in bold.

### Survival analysis

**Figure 4:**
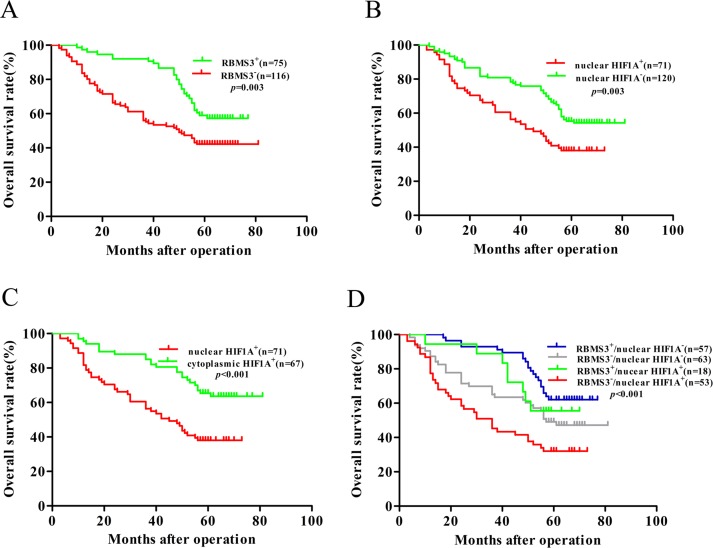
Kaplan–Meier survival analysis with Log-Rank test for the OS of 191 GC **A.** The OS of patients with RBMS3^−^ and RBMS3^+^. **B.** The OS of patients with nuclear HIF1A^+^ and nuclear HIF1A^−^. **C.** The OS of patients with nuclear and cytoplasmic HIF1A^+^. **D.** The OS of patients with subgroups stratified to RBMS3/HIF1A expressions.

Next, we evaluated the prognostic values of RBMS3 and HIF1A expression in patients with GC. Kaplan–Meier survival analysis confirmed that RBMS3 and nuclear HIF1A expression were significantly correlated with clinical prognosis. The patients who were RBMS3^−^ had worse OS [median 50 months, mean 48.953 ± 2.793 months] than those who were RBMS3^+^ [median 58 months, mean 63.193 ± 2.160 months, *p*=0.003, Figure 4A]. Similarly, the patients who were “nuclear HIF1A^+^” exhibited shorter OS [median 45 months, mean 44.099 ± 3.086 months] than “nuclear HIF1A^−^” patients [median 58 months, mean 60.408 ± 2.349 months, *p*=0.003, Figure 4B]. Moreover, “nuclear HIF1A^+^” patients had a worse OS [median 45 months, mean 44.099 ± 3.086 months] than “cytoplasmic HIF1A^+^” patients [median 64 months, mean 65.246±2.850 months. *p*<0.001, Figure 4C]. Together, our result demonstrates that RBMS3 and nuclear HIF1A are significantly associated with the clinical prognosis of GC.

Based on the above results, we further explored the relationships between the prognosis of GC patients and different combinations of RBMS3 and nuclear HIF1A expression. The combinations were: group 1, RBMS3^+^/nuclear HIF1A^−^; group 2, RBMS3^−^/nuclear HIF1A^−^; group 3, RBMS3^+^/nuclear HIF1A^+^; group 4, RBMS3^−^/nuclear HIF1A^+^. Kaplan-Meier analysis showed statistically distinct survival patterns among the four subgroups (Figure 4D, *p*<0.001). The RBMS3^+^/nuclear HIF1A^−^ patients had the most favorable prognosis [median 64 months, mean 65.109 ± 2.290 months], whereas the RBMS3^−^/nuclear HIF1A^+^ patients had the poorest prognosis [median 36 months, mean 39.415 ± 3.630 months, *p*<0.001]. These results indicate that combined expression of RBMS3 and nuclear HIF1A is a more reliable predictor of GC prognosis, than RBMS3 expression or nuclear HIF1A expression alone.

Next, we tested the possibilities of RBMS3 and HIF1A being the independent prognostic factors for GC. The univariate Cox regression analysis picked out the factors that were significantly associated with OS. They were tumor size, differentiation, depth of invasion, lymph node metastasis, TNM stages, RBMS3 and nuclear HIF1A expression (Table 3). Then, the multivariate Cox regression analysis was performed on these factors. It further supported the factors of differentiation, depth of invasion, lymph node metastasis and RBMS3 expression to be the independent prognostic factors (Table 3). In conclusion, our analysis indicate that the differentiation and depth of invasion of GC, together with lymph node metastasis levels and RBMS3 expression in GC could be the independent prognostic factors for GC.

**Table 3:** Univariate and multivariate analysis of the correlation between clinicopathological parameters and prognostic significance of GC patients

Variables	Univariate analysis	*p* value	Multivariate analysis	*p* value
	HR(95%CI)		HR(95%CI)	
Gender (male vs. female)	0.863(0.537-1.388)	0.543		NA
Age (y) (<61 vs. ≥61)	1.002(0.675-1.498)	0.991		NA
Tumor diameter (cm) (<6 vs. ≥6)	0.527(0.354-0.785)	**0.002**	0.710(0.472-1.067)	0.1
Differentiation (high/moderate vs. low/undifferentiated)	2.122(1.297-3.471)	**0.003**	1.785(1.074-2.965)	**0.025**
Location (upper vs middle vs. lower)	0.864(0.685-1.090)	0.217		NA
Depth of invision (T1/TI vs. T3/T4)	9.370(2.965-29.607)	**1.38E-04**	6.203(1.824-21.098)	**0.003**
Lymph node metastasis (yes vs. no)	3.763(2.095-6.759)	**9.16E-06**	3.451(1.590-7.492)	**0.002**
TNM stages (I/II vs. III/IV)	3.026(1.865-4.910)	**7.37E-06**	0.826(0.421-1.620)	0.577
RBMS3 (positive vs. negative)	0.534(0.349-0.818)	**0.004**	0.531(0.342-0.825)	**0.005**
Nuclear HIF1A (positive vs. negative)	1.787(1.199-2.664)	**0.004**	1.437(0.951-2.172)	0.085

Note: Variables with *p* values more then 0.05 in the univariate models were not adapted (NA) in the multivariate analysis. *p*<0.05 was defined statistically significant and was given in bold. CI: confidence interval. HR: Hazard ratio.

### RBMS3 regulates GC cells proliferation *in vitro*

**Figure 5:**
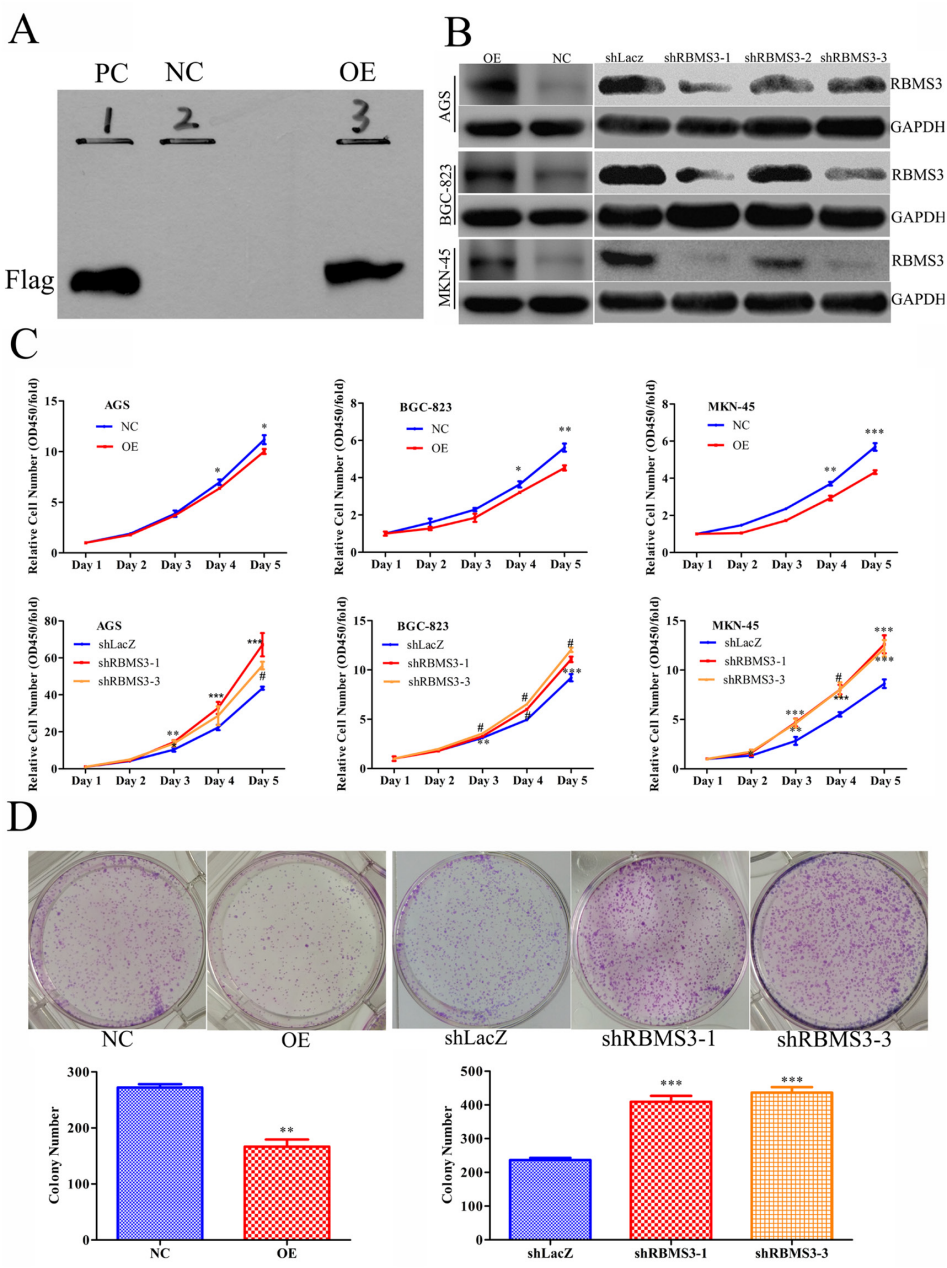
RBMS3 inhibited GC cell growth *in vitro* **A.** Western blot validation for 293 T cells with RBMS3 overexpression (OE) and negative control (NC), and PC as positive control. **B.** The expression of RBMS3 in AGS, BGC-823 and MKN-45 GC cell lines after lentivirus infection. **C.** Cell proliferation curve for cells with RBMS3 overexpression and knockdown. **D.** Colony formation for cells with RBMS3 overexpression and knockdown. Values are means ± SD. *, *p*<0.05; **, *p*<0.01; ***, *p*<0.001. #, *p*<0.0001.

Given that RBMS3 is significantly down-regulated in GC, and the close relationship between RBMS3 expression and clinical prognosis, we inferred that RBMS3 might inhibit GC cells growth. To confirm our hypothesis, we first chose GC cell lines AGS, BGC-823 and MKN-45 for *in vitro* study as they have the lowest mRNA expression levels of endogenous RBMS3 compared with other GC cell lines (date not shown). Then, we induced RBMS3 by lentivirus, and increased RBMS3 in 293T cells was confirmed by western blotting (Figure 5A). RBMS3 stably overexpressed or silenced AGS, BGC-823 and MKN-45 cells were established by lentiviruses infection, while the empty vector (NC) or shRNA targeting LacZ (shLacZ) served as control groups respectively. Three lentiviral shRNA constructs (shRBMS3-1, shRBMS3-2 and shRBMS3-3) designed against different regions of RBMS3 were introduced separately into AGS, BGC-823 and MKN-45 cells via infection. Western blot showed shRBMS3-1 and shRBMS3-3 markedly reduced the level of RBMS3 expression compared with shRBMS3-3 (Figure 5B). Therefore, we used shRBMS3-1 and shRBMS3-3 for our downstream applications.

Next, we examined the effects of RBMS3 on the proliferation of AGS, BGC-823 and MKN-45 cells using MTT assay. As shown in Figure 5C, overexpression of RBMS3 significantly inhibited the proliferation of AGS, BGC-823 and MKN-45 cells whereas the blockade of endogenous RBMS3 expression markedly promoted cell growth. Furthermore, we performed clonogenic assays on MKN-45 cell, the number and size of the colonies were remarkably decreased in MKN-45 cells with RBMS3 overexpression, whereas RBMS3 depletion significantly enhanced colony formation (Figure 5D). These results suggest that RBMS3 inhibit the proliferation of GC cells *in vitro,* and depletion of it may promote GC cell growth.

### RBMS3 inhibits cell cycle progression in GC cells

**Figure 6:**
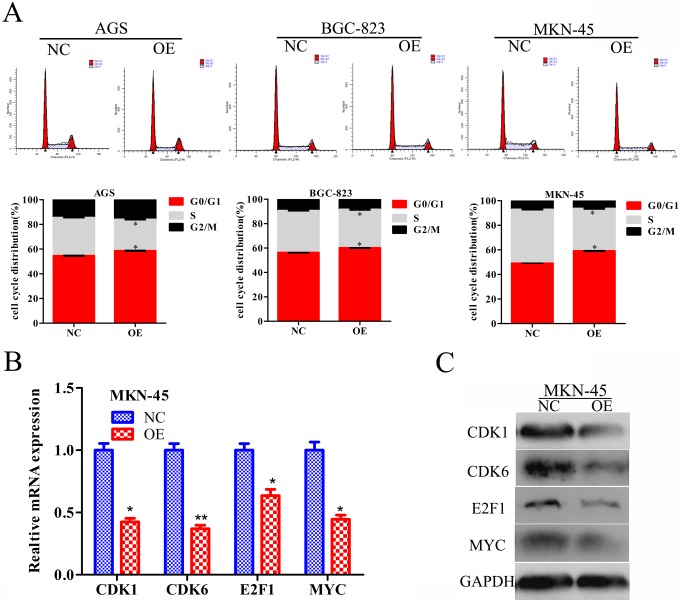
RBMS3 regulates G1/S phase progression of GC cell cycle **A.** The cell cycle distribution of AGS, BGC-823 and MKN-45 GC cells after RBMS3 overexpression. Values are means ± SD. *, p<0.05. The level of cell cycle-related genes in the G1/S transition were measured by q-PCR **B.** and western blot **C.** assays. GAPDH served as an endogenous control.

To understand the mechanism underlying the inhibition of cell proliferation, we performed flow cytometry to analyse whether the cell cycle distribution was altered after RBMS3 overexpression in AGS, BGC-823 and MKN-45 cells. Cell cycle analysis showed that overexpression of RBMS3 notably increased the percentage of the G0/G1 phase and decreased that of S phase (Figure 6A). We then investigated the effects of RBMS3 on the expression of cell cycle-related genes. q-PCR and western blot showed that the mRNA and protein expression levels of CDK1, CDK6, E2F1 and MYC were down-regulated upon RBMS3 overexpression in MKN-45 cell (Figure 6B and 6C). Taken together, these results reveal that RBMS3 overexpression inhibits the GC cell cycle progression, at least in part, by regulating cell cycle-related proteins.

### Correlation between MVD and RBMS3, HIF1A expression

**Figure 7:**
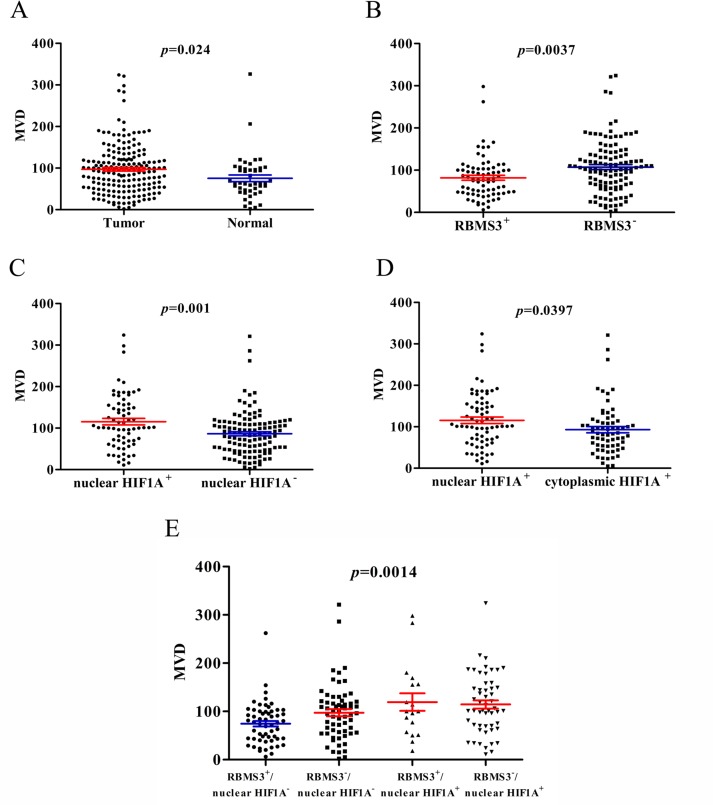
MVD counting of tumor and normal tissues **A.** MVD counting in tumor compared with normal tissues. **B.** MVD counting in tumor tissues with RBMS3^+^ and RBMS3^−^. **C.** MVD counting in tumor tissues with nuclear HIF1A^+^ and nuclear HIF1A^−^. **D.** MVD counting in tumor tissues with nuclear HIF1A^+^ and cytoplasmic HIF1A^+^. **E.** MVD counting in tumor tissues with subgroups of RBMS3/nuclear HIF1A.

Previous reports showed that the RBMS3 and HIF1A expression were closely related to MVD in some tumors [13, 16], but were unclear in GC. Therefore, we checked the correlation between MVD and RBMS3, HIF1A expression in our GC cohort. MVD were recorded by counting the CD34 staining. Generally, the mean MVD in GC was significantly higher than that in normal controls (97.29 ± 4.307, 75.46 ± 7.961, t=2.272, *p*=0.024, Figure 7A). For RBMS3, the MVD in RBMS3^+^ group (81.85 ± 5.814) were lower than that in RBMS3^−^ group (107.3 ± 5.848, t=2.939, *p*=0.0037, Figure 7B) in GC samples.

In addition, nuclear HIF1A^+^ group (115.6 ± 7.800) showed higher MVD than nuclear HIF1A^−^ group (86.46 ± 4.830, t=3.356, *p*=0.001, Figure 7C) in GC samples. To further evaluate the role of nuclear and cytoplasmic HIF1A^+^ respectively, we compared the MVD in two GC subgroups. Interestingly, nuclear HIF1A^+^ (115.6 ± 7.800) group had higher MVD than cytoplasmic HIF1A^+^ group (93.10 ± 7.476, t=2.077, *p*=0.0397, Figure 7D). The results implied that nuclear HIF1A^+^ played a more important role in MVD formation than cytoplasmic HIF1A^+^ in GC samples. Furthermore, when we divided the GC samples into four subgroups as mentioned before, the RBMS3^−^/nuclear HIF1A^+^ subgroup had higher MVD than the RBMS3^+^/nuclear HIF1A^−^ subgroup (*p*<0.001, Figure 7E). In summary, our results show that the MVD is closely related to RBMS3 and nuclear HIF1A expression in GC.

### RBMS3 regulates GC cells angiogenesis *in vitro*

**Figure 8:**
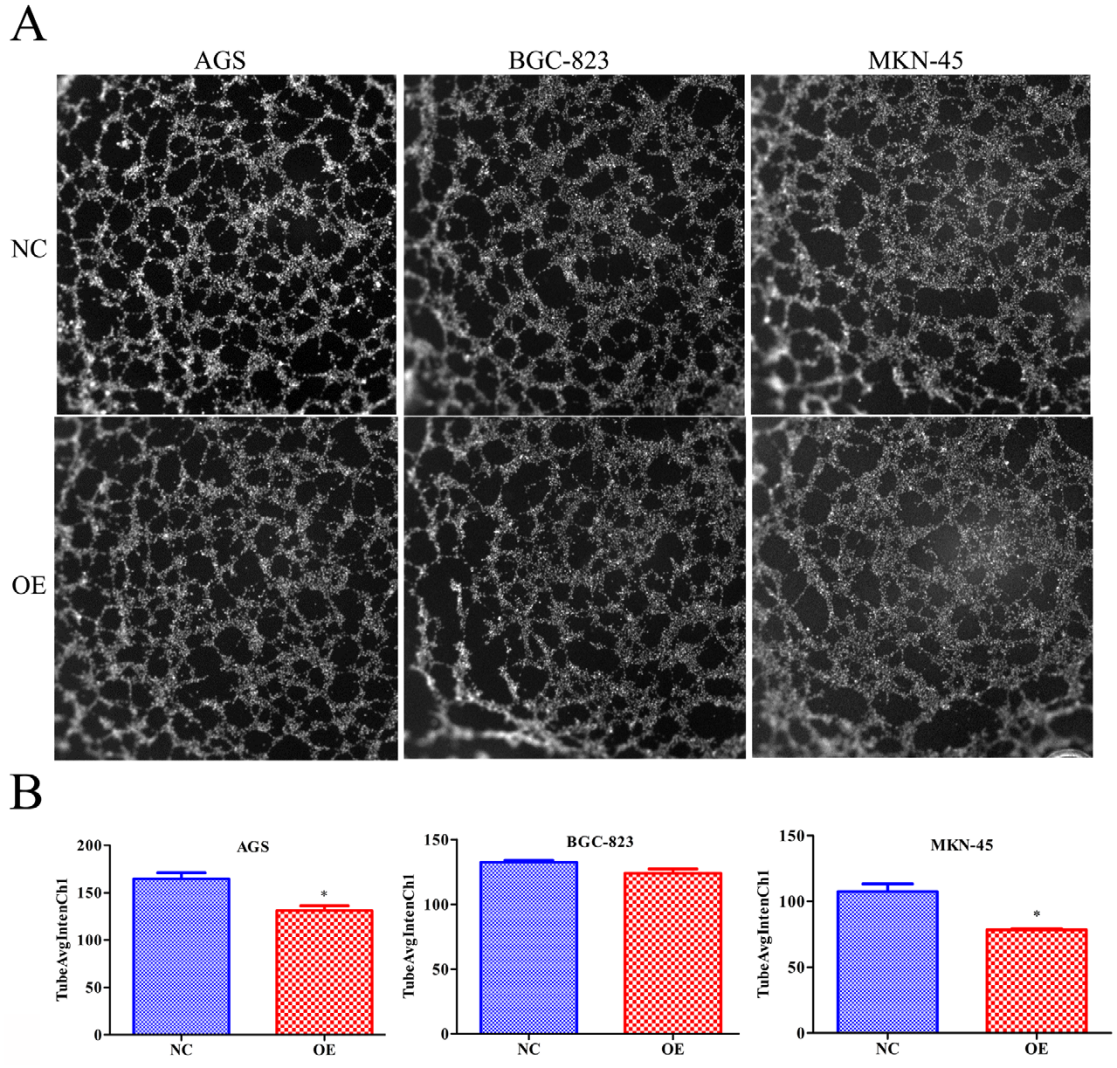
Overexpression of RBMS3 inhibited the angiogenesis of gastric cancer cell **A.** Images of endothelial cell tube formation assay. **B.** TubeAvgIntenCh1 analysis of endothelial cell tube formation assay. Values are means ± SD. *, *p*<0.05.

To further evaluate whether RBMS3 regulates the angiogenisis of GC, we performed endothelial cell tube formation assays *in vitro*. As shown in Figure 8A and 8B, the number of complete tubes induced by the conditioned medium of RBMS3-overexpression AGS and MKN-45 cells was significantly lower TubeAvgIntenCh1 than that of the negative control (Figure 8B, *p*=0.015, *p=*0.043, respectively). However, for BGC-823 cell line, although it did not reach statistical significance, a clear trend toward RBMS3-overexpression inhibiting angiogenesis was also observed (*p*=0.085). In summary, our data support that RBMS3 might suppress the angiogenesis of GC cells *in vitro*.

## DISCUSSION

Deletion of multiple regions on the short arm of chromosome 3 (3p) is one of the most frequent genetic alterations in many human solid tumors including GC. RBMS3, located at 3p24, have been reported as a TSG. Previous studies showed that RBMS3 was mainly found in nuclei and its low expression in nuclei predicted unfavorable prognosis for patients with LSCC, NPC and ESCC [9, 10, 12]. However, we found RBMS3 staining was only seen in cytoplasm in our cohorts. This discrepancy is possibly because of the various functions of RBMS3 in different malignancies. What's more, our finding consisted with the conclusion of Penkov [11], whose study demonstrated RBMS3 accumulation in the cytoplasm and absence from the nuclei. Hence, our findings agreed that RBMS3 might function in the cytoplasm of GC cell.

It was reported that down-regulation of RBMS3 was associated with poor prognosis and tumor angiogenesis in some cancers [9, 10]. In this study, we investigated the effect of RBMS3 expression on prognosis and tumor angiogenesis in GC and found RBMS3^−^ GC patients were significantly related to poorer prognosis and higher tumor angiogenesis compared with RBMS3^+^ GC patients, which was consistent with the previous studies [9, 10, 12]. Moreover, our multivariate Cox analysis further demonstrated that RBMS3 could be an independent prognostic factor for GC, which was further verified by our *in vitro* experiments.

Hypoxia is a hallmark of tumor microenvironment and is associated with angiogenesis, invasion, metastasis, and treatment resistance [22–24]. Therefore, the adaptation to hypoxia is essential to the survival of cancer cells [25]. Tumor angiogenesis was correlated with metastasis and poor prognosis in some cancers [26–28]. As was reported, HIF1A played a major role in response to hypoxia and was also mainly responsible for the “angiogenic switch”. HIF1A was commonly found to locate in the nucleus of most cancers and absented in normal controls [15–17]. However, recent findings had revealed the positivity of nuclei HIF1A in some normal human tissue types [29, 30], suggested that nuclear HIF1A had a physiological role in the normal tissues. In our study, HIF1A staining was detected in both nuclei and cytoplasms of cancer and normal tissues, and nuclear HIF1A staining was significantly increased in cancer compared with normal controls. But there was no difference between the two groups in cytoplasmic HIF1A staining. Since nucleic HIF1A determined the functional activity of the HIF1A complex [31, 32], we investigated the effect of cytoplasmic HIF1A and nucleic HIF1A on the prognosis of GC patients respectively by measuring MVD and survival analysis. MVD in nucleic HIF1A^+^ were significant higher compared with cytoplasmic HIF1A^+^ in cancer tissues. Moreover, GC patients with nucleic HIF1A^+^ had poorer OS than those with cytoplasmic HIF1A^+^. These results showed that the cytoplasmic and nucleic expression of HIF1A might exert different roles in during progression and angiogenesis of GC. So it is critical to analyze the effect of cytoplasm HIF1A and nucleic HIF1A on the prognosis of GC patients separately.

Compared with RBMS3, the prognostic value of HIF1A in GC is still controversial. Agreed with Sumiyoshi *et al* [33], our discovery showed that HIF1A^+^ GC patients had a shorter OS than HIF1A^−^ GC patients. However, the findings were not consistent with the results of Kolev *et al* [34] and Urano *et al* [25], which showed that overexpression of HIF1A had no association with patients prognosis. Since neither studies separately investigated the effect of cytoplasmic and nucleic HIF1A expression on patients prognosis, it was possible that the actual contribution of nucleic HIF1A was masked by cytoplasmic HIF1A as we showed in this study. Furthermore, our Kaplan-Meier analysis revealed that patients of the RBMS3^−^/nuclear HIF1A^+^ subgroup showed the worst OS, whereas the RBMS3^+^/nuclear HIF1A^−^ subgroup exhibited the best OS. These results suggested that the combination of RBMS3 and nucleic HIF1A could be a key molecular prognostic indicator for GC patients.

In the family of MSSP, RBMS1 is another important member that inhibits HIF1A expression and induces its degradation [11, 35–37]. In addition, RBMS1 promoted HIF1A translocation from nuclei to cytoplasm and decreased microvessel density [38–40]. These evidences prompted us to assess the association between RBMS3 and HIF1A in tumors. Spearman's rank correlation analysis showed that RBMS3 negatively correlated with nuclear HIF1A and positively correlated with cytoplasmic HIF1A in GC. The finding indicated that RBMS3 might also regulate the cellular localization of HIF1A and was consistent with the function of RBMS1. In addition, the MVD in RBMS3^−^/nuclear HIF1A^+^ subgroup were higher than other subgroups, which were associated invasion, metastasis and poorer prognosis [26–28].

In conclusion, we showed that RBMS3 could be an independent prognostic factor for GC. In addition, we implied that RBMS3 might modulate the location of HIF1A and associate with tumor angiogenesis. Furthermore, the RBMS3^−^/nuclear HIF1A^+^ patients had the poorest prognosis, which indicated that combined expression of RBMS3 and nuclear HIF1A was a more reliable predictor of GC prognosis, than RBMS3 expression or nuclear HIF1A expression alone. This finding implicated that down regulation of RBMS3, along with up regulation of nuclear HIF1A could act as a novel therapeutic molecular target for GC and might promote angiogenesis in GC. From our study, we call for further researches of the molecular mechanisms of HIF1A location, which will likely provide new insights into the pivotal function of RBMS3 in cancer biology and should provide a novel approach to the treatment of GC.

## MATERIALS AND METHODS

### Patients and tissues specimens

In our study, a total of 191 formalin-fixed, paraffin-embedded GC and 46 randomly selected normal gastric tissues were gathered for the tissue microarray (TMA) from the department of general surgical of the First Affiliated Hospital of Anhui Medical University (Hefei, China) from December 2006 to September 2008. To get the complete basic clinical data, all the patients had regular follow-up visits with every 3 months for the first 2 years after surgery, and every 6 months for the next several years. Complete follow-up was updated until November 2013. The immunohistochemical stainings was analyzed by experienced pathologists. All the patients' pathological features were also confirmed by two experienced pathologists, and pathological TNM staging was analyzed depended on 2010 the 7th edition of the American Joint Committee on Cancer (AJCC) TNM classification criteria. The clinicopathologic characteristics of the TMA were described in Table 1. There were 144 males and 47 females, with a mean age of 60.36 years and a median age of 61 (range, 29 to 87 years). As required, all of these patients were absence of any anti-cancer treatment before surgery. To further explore the different expression of protein and mRNA of RBMS3 and HIF1A between GC and normal gastric tissues. The fresh cancer tissues and matched normal tissues (at least 5 cm distant from the tumor edge) were immediately frozen in liquid nitrogen and stored at −80°C to extract protein and RNA. Written informed consents were provided by all patients. This study obtained approval from the Institute Research Ethics Committee of the First Affiliated Hospital of Anhui Medical University.

### RNA preparation, reverse transcription and Real-time qPCR

Total RNA was extracted from fresh frozen tissues and cell lines using TRIzol Reagent (Invitrogen), and reverse transcription (RT) using ReverTra Ace qPCR RT Master Mix (Toyobo) according to the manufacturer's protocol. qPCR was performed using ABI 7900HT Sequence Detection System (Applied Biosystems, CA, USA) in the presence of SYBR-Green dye (Toyobo, Osaka, Japan). The primers were as follows: RBMS3, forward 5′-GGTAGCATCTCTCAAGGCAAAT-3′, reverse 5′-CATGTCCAAAGGGTTTCAGCA-3′; HIF1A: forward 5′-ATCCATGTGACCATGAGGAAATG-3′, reverse 5′-TCGGCTAGTTAGGGTACACTTC-3′; Glyceraldehyde-3- phosphate dehydrogenase (GAPDH) as the internal control: forward 5′-ATCAAGAAGGTGGTGAAGCAGG-3′, reverse 5′-CGTCAAAGGTGGAGGAGTGG-3′. The amplification was done in a total volume of 10 μl with the following steps: denaturation program at 95°C for 5 min, followed an amplification and quantification program for 40 cycles at 95°C for 15 s and 60°C for 45 s. All experiments were done in triplicates. A melting curve analysis was used to check the specificity of amplification. GAPDH was used as the internal control. The relative expression of each sample was calculated using the 2^−ΔΔCT^ method.

### Western blot

Total proteins were extracted from fresh frozen tissues and cell lines by RIPA lysis buffer. The protein concentration of the supernatant was detected by the BSA Protein Assay Kit (Beyotime institute of Biotechnology, Jiangsu, China). The protein samples were separated on 8% or 10% sodium dodecyl sulfate polyacrylamide gel electrophoresis and electrotransferred to nitrocellulose membranes (Millipore, Billerica, MA). After blocking with 5% nonfat milk dilution with TBST (tris-buffered saline with tween-20) for 1 h at room temperature, the membranes were incubated with rabbit anti-RBMS3 antibody (1:1000; Abcam) and rabbit anti-HIF1A antibody (1:1500; Abcam) at 4°C overnight. After washing 3 times with TBST per 10 min, the membranes were then incubated with horseradish peroxidaselabeled anti-rabbit IgG as the secondary antibody (Epitomics) at room temperature for 60 min. Afterwards, after 3 times washing with TBST per 10 min, the membranes were detected with the enhanced chemiluminescence system. Anti-GAPDH antibody (1:3000; vazyme) was used as a loading control.

### Immunohistochemistry (IHC)

Before IHC, H&E-stained slides were screened to identify optimal intratumoural tissue for analysis. Multiple 4-μm-sections were cut with a Micron microtome, and then cut section were baked at 63°C for 1 h, deparaffinized with xylenes, and rehydrated in graded ethanol to distilled water. Antigen retrieval was performed by placing the sections in 0.01 M citrate buffer pH 6.0 in pressure cooker for 5 min at 120°C. After antigen retrieval. The endogenous peroxidase activity of the sections were quenched by 3% hydrogen peroxidase (H_2_O_2_) in methanol. To inhibit non-specific antigen-antibody reactivity in the immunohistochemically stained sections by 1% bovine serum albumin. Then the slides were incubated with primary antibody, anti-RBMS3 (1:25, Abcam), anti-HIF1A (1:300, Abcam) and anti-CD34 (1:400, Abcam) overnight at 4°C followed by washing. Thereafter, the sections of bound primary antibody were detected by horseradish peroxidase-labelled anti-rabbit IgG secondary antibodies at room temperature for 30 min followed by washing and visualized using an autostainer link instrument and proceed with staining. As negative staining controls, the primary antibodies were treated with normal rabbit serum. The results of immunohistochemistry staining score were detected by two independent experienced pathologists who without prior knowledge of clinical pathologic information for this patients. We classified immunoreactivity locating in cells as follows: nucleus only, cytoplasm only, nucleus and cytoplasm and both negative. We categorized these subgroups were nucleus-negative and -positive, and cytoplasm-negative and -positive. According to the dominant staining intensity of GC mucosa cells and normal mucosa cells, the scored were classified in four grades by the percentage of stained (0 points for no cells stained, 1 points for <25%, 2 points for 25-75%, and 3 points for >75% of cells stained), and the staining intensity of immunoreactivity was graded on a scale of 0 to 3 four grades (intensity scores): negative (0), weak (1), moderate (2) and strong (3). The immunoreactivity score (IRS) was defined as multiplication of both parameters. Specimens were scored as follows: negative or weak (-, IRS = 0 ~ 2), positive (+, IRS = 3 ~ 9).

### MVD counting

Microvessels were recorded by counting the CD34 stained with the generally accepted criteria performed by Weidner et alz [26]. Any stained endothelial cells or endothelial cell clusters were separated from other nearby microvessels. The thickness of vessel wall over 2.75 μm or vessels with thick muscular walls were excluded from the count. The “hot spots” were confirmed by the lower magnification objectives (100×), which were described as the areas with the highest number of microvessels. Then, microvessels were counted within the “hot spot” by the high power (200×, the surface area of every visual field was 0.5 mm^2^). The finally MVD counting was recorded by the average of 3 high power visual field.

### Cell culture and lentivirus infection

AGS, BGC-823 and MKN-45 human GC cell lines, and 293T human embryonic kidney cell line were obtained from Genechem (Shanghai, China). All cell lines were cultured in RPMI1640 medium (Gibco, USA) supplemented with 10% fetal bovine serum (FBS, Gibco, USA), 100 IU/ml penicillin and 100 μg/ml streptomycin, and incubated at 37°C in a humidified incubator containing 5% CO2. RBMS3 overexpression lentiviral vector GV365 (OE, Ubi-RBMS3- 3FLAG- CMV-EGFP) and the negative control GV365 (NC, Ubi-MCS-3FLAG-CMV-EGFP) were obtained from Genechem (Shanghai, China). To interfere RBMS3 expression, the shRNA target sequences for human RBMS3 were as follows: shRBSM3-1 (clone ID: TRCN0000153312): 5′-GCAGATGAATCACCTTTCGTT-3′; shRBSM3-2 (clone ID: TRCN0000152525): 5′-GACATCTATCAC GCCATTCAT-3′; shRBSM3-3 (clone ID: TRCN0000152012): 5′-CACAAATCAGTGCAAAG GTTA-3′. A shLacZ with the target sequence of 5′-GGATCAGTCGCTGATTAAA-3′ was severed as a control [41]. The oligonucleotides were inserted into the pLL3.7 lentiviral vector. The procedures of packaging and infection of lentivirus were conducted as previously described [42]. Overexpression and knockdown of target genes efficacy were tested by Western blot analysis.

### Cell proliferation assay and clonogenic assay

Cell proliferation assays were employed to evaluate the effects of RBMS3 on proliferation capabilities of GC cell lines. In brief, the different lentivirus infection cells were seeded into 96-well plates (about 2000 cells/well) in sextuple and cell proliferation rates were evaluated by MTT (Genview) assay at different time points according to the manufacturer's instructions. Each assay was performed at least in three times.

Clonogenic assays were conducted to investigate the effect of RBMS3 on cell growth and proliferation capabilities of GC cell lines. In short, the different lentivirus infection cells were seeded into 6-well plates and the medium was replaced every three days, and cells were incubated for 14 days before being fixed with ice-cold methanol and stained with Giemsa's stain, and the number of colonies (>50 cells/colony) was then counted. Each assay was repeated at least in three times.

### Cell cycle analysis

Cell cycle distribution was analyzed as described previously [43]. Briefly, lentivirus infection cells were incubated with propidium iodide (PI, 10 μg/ml) containing RNase at 4°C for 30 min in the dark. The fractions of viable cells in G0/G1, S and G2 phases of cell cycle were measured with a FACS flow cytometer and Cell Quest FACS system (Becton-Dickinson).

### Endothelial cell tube formation assay

The tube formation assay was performed as described previously [44]. Briefly, HUVECs (2 × 10^4^/well) were seeded into 96-well plates coated with Matrigel Basement Membrane Matrix (70μL, BD Biosciences) and then incubated at 37°C for 30 minutes to polymerize. HUVECs were harvested after trypsin treatment and seeded in wells with different conditioned media from RBMS3 overexpression group and NC group. Then 96-well plates were incubated at 37°C in a 5% CO2 atmosphere for 20 hours with above these conditioned medium. The cultures were stained with Cellomics Cytoskeletal Rearrangement Kits (Thermo Fisher Scientific) according to the protocol by the company and analyzed with Cellomics (Thermo Fisher Scientific). The degree of tube formation was assessed as average intensity of all Pixels with channel1 object (tube) mask (TubeAvgIntenCh1, Thermo Fisher Scientific provided).

### Statistical analysis

All statistical analyses were performed by SPSS 16.0 software (SPSS, Inc.Chicago, IL). The relations between RBMS3, HIF1A expression and clinicopathologic characteristics were assessed by the Pearson χ^2^ test. The correlation between RBMS3 and HIF1A was analyzed using the Spearman's rank test. The differential expression of mRNA between in fresh frozen GC and paired normal tissues were analyzed by paired-samples t test. Overall survival (OS) were analyzed by Kaplan-Meier method, and the differences significance of survival curves was displayed by the log-rank test. Using the Cox proportional hazards model, multivariate analysis was only performed on the significance of variables in univariate analysis. All statistical tests were two sided. *p*<0.05 were considered statistically significant.
